# Functional Magnetic Stimulation in the Management of Lower Urinary Tract Dysfunction in Children with Asperger Syndrome: A Case Report

**DOI:** 10.3390/children12101340

**Published:** 2025-10-05

**Authors:** Edva Anna Frunda, Orsolya Katalin Ilona Mártha, András Kiss, Árpád Olivér Vida, Tibor Lóránd Reman, Raul-Dumitru Gherasim, Veronica Maria Ghirca, Bogdan Călin Chibelean, Daniel Porav-Hodade, Carmen Viorica Muntean

**Affiliations:** 1 Institution Organizing University Doctoral Studies (I.O.S.U.D.), George Emil Palade University of Medicine, Pharmacy, Science, and Technology of Târgu Mureș, 540139 Târgu Mureș, Romania; anna.frunda@gmail.com; 2 Department of Urology, George Emil Palade University of Medicine, Pharmacy, Science, and Technology of Târgu Mureș, 540139 Târgu Mureș, Romania; arpad.vida@umfst.ro (Á.O.V.); tibor.reman@umfst.ro (T.L.R.); raul-dumitru.gherasim@umfst.ro (R.-D.G.); maria.ghirca@umfst.ro (V.M.G.); calin.chibelean@umfst.ro (B.C.C.); daniel.porav-hodade@umfst.ro (D.P.-H.); 3 Department of Urology, Târgu Mureș Clinical Hospital, P-ța Bernády György, Nr. 6, 540072 Târgu Mureș, Romania; 4 Department of Pediatrics—Heim Pál, Semmelweis University, Baross utca 22, 1083 Budapest, Hungary; kissandras@heimpalkorhaz.hu; 5 Department of Pediatrics, George Emil Palade University of Medicine, Pharmacy, Science, and Technology of Târgu Mureș, 540139 Târgu Mureș, Romania; munteancarmen@umfst.ro; 6 Department of Pediatrics, County Emergency Hospital of Târgu Mureș, Str. Gh. Marinescu, Nr. 50, 540136 Târgu Mureș, Romania

**Keywords:** Asperger syndrome, LUTS, uroflowmetry, urinary retention, urinary incontinence, FMS

## Abstract

**Background/Objectives:** A variant of autism spectrum disorder (ASD) known as Asperger syndrome (AS) shows increasing incidence worldwide, affecting between 0.02% and 0.03% of children. Patients display abnormal conduct, are limited in social interaction and communication, and are more often affected by micturition disorders, incontinence, and voiding symptoms than typically developing children. **Methods:** The present study aimed to review the literature related to the current management of lower urinary tract conditions in children with Asperger syndrome and to present a case of a 14-year-old girl with ASD, with characteristic impairments, including communication challenges, stereotyped, repetitive behaviors, and chronic constipation with concomitant bladder dysfunction, presenting recurrent urinary tract infections (UTIs) and lower urinary tract symptoms (LUTS), including voiding and filling storage symptoms. For the AS, she was treated with a selective serotonin reuptake inhibitor (Sertraline). An abdominal ultrasound, PLUTTS—pediatric lower urinary symptoms scoring (21); QL-quality of life (3); voiding diary; and uroflowmetry were performed, revealing an incomplete urinary retention (incomplete bladder emptying of 120 mL), a prolonged and interrupted curve, a maximum urinary flow rate (Qmax) 7 mL/s, and a UTI with Enterococcus. **Results:** Besides psychiatric reevaluation and antibiotic therapy, functional magnetic stimulation (FMS) sessions were performed. After eight sessions (20 min, 35 MHz, every second day), the ultrasound control and the uroflowmetry showed no residual urine, and the Qmax was 17 mL/s. The curve continued to be interrupted: PLUTSS-11, QL-1. FMS was continued at two sessions per week. At the 3-month follow-up, no residual urine was detected, and Qmax reached 24 mL/s. **Conclusions:** ASD is an incapacitating/debilitating condition that significantly impairs social functioning. In many cases, in addition to psychological symptoms, other conditions such as LUTS and constipation may coexist. Antipsychotics and antidepressants are frequently prescribed for these patients, often leading to various side effects, including micturition disorders. Therefore, screening for LUTS is recommended, and, if indicated, treatment—especially non-pharmacological and non-invasive approaches, such as FMS—should be considered.

## 1. Introduction

ASD refers to a group of neurodevelopmental disorders, which includes autism, AS, and pervasive developmental disorder—not otherwise specified (PDD-NOS). The occurrence of ASD has been increasing worldwide, with a current estimated prevalence of 1 in 36 children [1,2]. ASD is characterized by severe issues with interpersonal, verbal, and nonverbal communication, as well as restricted and repetitive patterns of behavior and activities (Moylan) [3,4]. AS very often co-occurs with other neuropsychiatric problems, such as obsessive-compulsive behavior, sleep disorder, Tourette syndrome, attention deficit hyperactivity disorder, and anxiety disorder. The published literature indicates a higher prevalence of incontinence in children with ASD compared to typically developing children. Filling disorders, incontinence, and retention are also reported as side effects of medication in ASD [3,4,5]. Among the few studies conducted on children with ASD, gastrointestinal symptoms have received more attention than LUTS. Children with ASD who present with rigidities, anxiety, or sensory preferences may establish a pattern of holding urine and stool, which places them at high risk of developing bladder bowel dysfunction (BBD) [6,7,8]. BBD, despite being common, is often unrecognized in children with ASD. The literature review indicates a complex interplay of factors such as brain connectivity changes, the maturational delay of bladder function, cognitive rigidities, and psychosocial stressors, which may collectively predispose some children with ASD to develop BBD [9,10]. Simple interventions, such as parental education, the use of a bladder and bowel diary, and constipation management, may help to alleviate symptoms [8]. Unfortunately, there is no specific medical therapy that can effectively cure all autism-related symptoms. However, medications may be used as adjuvant therapy. Among the drugs used in pharmacological treatment are psychostimulants, antipsychotics, antidepressants, and alpha-2 adrenergic receptor agonists. Among these, risperidone (an antipsychotic) and selective serotonin reuptake inhibitors (SSRIs), such as sertraline, fluoxetine, and citalopram, can help reduce irritability, depression, aggression, anxiety, or hyperactivity [11,12].

It is also well known that these therapies can have important side effects, including nausea, a loss of appetite, diarrhea, indigestion, increased sleepiness or insomnia, increased sweating, and sexual dysfunction. Additionally, they may impact micturition, leading to conditions such as increased urinary frequency (pollakiuria) and urinary incontinence. Despite the increasing incidence of these disorders, few studies have examined LUTS in children with Asperger syndrome. The evaluation, diagnosis, and treatment of lower urinary tract dysfunction (LUTD) and psychiatric disorders in children are difficult, time-consuming, and require a multidisciplinary approach involving professionals such as pediatricians, urologists, psychiatrists, and other specialists. Our study’s objective was to present our experience with FMS in the treatment of children’s micturition disorders associated with AS while also providing a systematic review of different treatment modalities in these cases [13].

## 2. Materials and Methods

**Figure 1:**
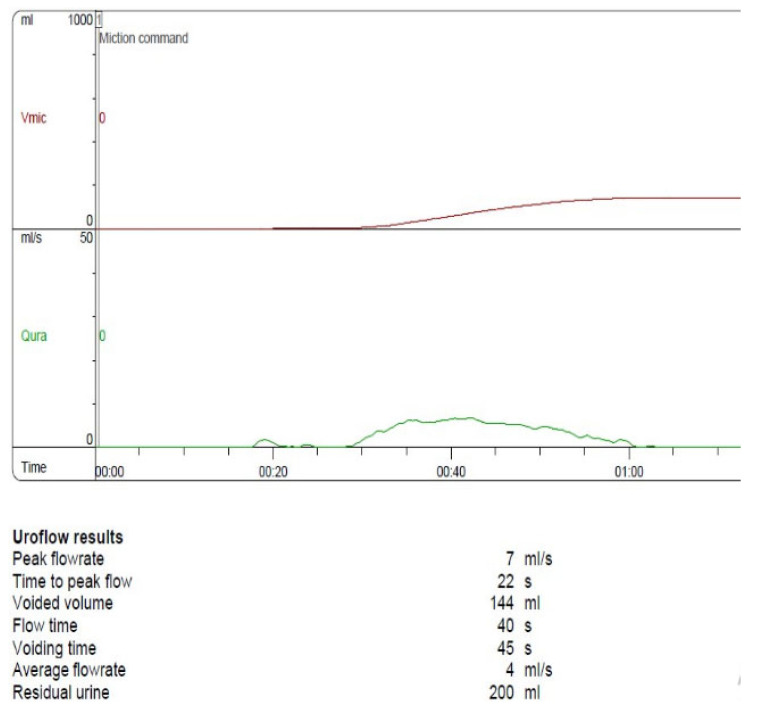
Uroflowmetry (Qmax 7 mL/s, PVR—120 mL) before treatment. Red line—V mic, Green line—Qmax.

A 14-year-old girl was referred for a urological examination due to recurrent urinary infection, LUTS, urge incontinence, and a slow, intermittent urinary flow. She was diagnosed with Asperger syndrome at the age of six and was undergoing treatment with Sertraline. In addition to the characteristic impairments of Asperger syndrome, including communication challenges, repetitive behaviors, and micturition disorders, she also experienced chronic constipation. PLUTTS was completed (score: 21, QL-3), and diagnostic investigations were performed, including an abdominal ultrasound and uroflowmetry. Findings revealed incomplete urinary emptying with a post-void residual volume of 120 mL, prolonged and interrupted flow curve, Qmax of 7 mL/s (Figure 1), and a urinary tract infection caused by Enterococcus faecalis. A three-day voiding diary recorded 11 micturitions/day, with a voided volume of 100–150 mL and mild urge incontinence.

A multidisciplinary team, including a pediatrician, psychiatrist, neurologist, and urologist, discussed the treatment strategy. The psychiatric specialist recommended the continuation of antidepressant therapy, while antibiotherapy and complex urotherapy—consisting of behavioral modifications and pelvic floor training—were initiated.

Based on the fact that FMS is an effective modality for the management of stress, urge and mixed urinary incontinence, fecal incontinence, pelvic floor muscle rehabilitation, and other voiding disorders, FMS using a Magneto Stym device was initiated, following the confirmation of a negative urine culture. The pulsed magnetic field generated by the device induces the muscles of the pelvic floor to contract without the need for any electrodes. FMS serves as an effective alternative to conventional electrical stimulation methods, offering several advantages. Magnetic fields are less painful, allow deep penetration into heterogeneous biological tissue, and do not require skin contact, aspects with great importance for children with AS.

Furthermore, FMS does not activate pain receptors on the skin surface, making it a more comfortable option compared to traditional electrical stimulation.

FMS treatment increases both the strength and endurance of the pelvic floor muscles. Additionally, it helps patients in learning how to properly perform muscle-strengthening exercises.

## 3. Results

**Figure 2:**
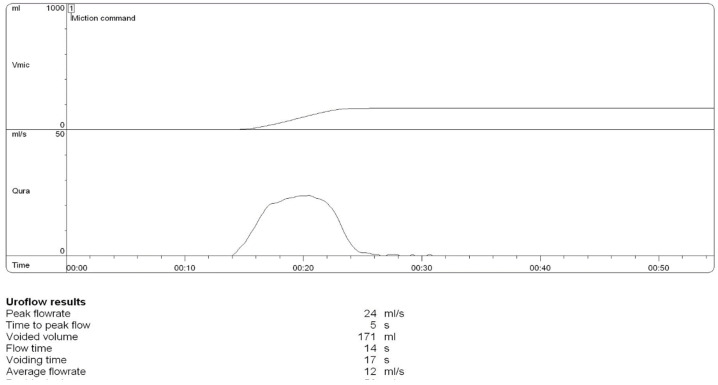
Uroflowmetry control after 3 months.

FMS was carried out very comfortably by the patient. After eight sessions (20 min, 35 MHz, every other day), starting with an empty bladder, the ultrasound and uroflowmetry control (Figure 2) revealed no residual urine, and the Qmax reached 17 mL/s. The curve remained interrupted, but no UTI was detected. The therapy continued at two sessions per week weekly, even when the bladder contained 150 mL of urine. A 3-month follow-up showed no residual urine and a very good Qmax of 24 mL/s.

FMS was continued after 3 months with weekly sessions lasting 20 min, with a volume of 150 mL urine in the bladder.

## 4. Discussion

The occurrence of AS has been increasing globally; like autism, it can neither be prevented nor treated [2]. Research on the relationship between ASD and LUTD remains limited. Existing studies suggest a higher prevalence of incontinence in children with ASD compared to their typically developing peers. Additionally, ASD-related symptoms have been observed in incontinent children [5,14,15]. Children with ASD who experience rigidities and anxiety may establish a pattern of urine and stool retention, developing BBD, incomplete emptying, and recurrent UTIs. These dysfunctions are common but often remain unrecognized and untreated, resulting in important complications. A recent review of the literature highlights a complex interplay of factors contributing to BBD, including brain connectivity alterations, delayed bladder function maturation, micturition issues, cognitive rigidity, and psychosocial stressors [8].

An Italian national survey on bladder and BBD in the ASD population showed that LUTS are very common/highly prevalent among young individuals with ASD with the prevalence of micturition disorders correlating with the severity of ASD [9]. It is obvious that LUTS and behavioral disorders in children are commonly concomitant/frequently coexist, making it challenging to treat one without addressing the other [6]. Children with ASD who experience rigidities, anxiety, or sensory preferences may establish a pattern of holding urine and stool/urine and stool retention. As a result, this complex psychiatric, medical disorder is frequently associated with LUTS, especially incontinence, urgency, and even postponement [8]. There is currently no consistent information on vesico-sphincter and bowel dysfunction specifically related to Asperger’s syndrome. Therefore, screening for micturition disorders in pediatric cases with ASD is recommended, and treatment of associated LUTD should be considered. The assessment of children and adolescents with AS requires an interdisciplinary team capable of addressing all aspects of this disease. As presented in our study, in order to recognize and prevent severe consequences of micturition disorders through the use of the LUTS Pediatric Questionnaire and performing urodynamic studies in cases of AS may be a valuable option [14,15].

Furthermore, there is no standardized protocol or a specific pharmacological prescription regarding the management of these dysfunctions. Urologists, pediatricians, and nephrologists should be aware of the frequent occurrence of micturition disorders and LUTS in patients with ASD [9]. The use of antidepressants and antipsychotic drugs can alleviate symptoms like irritability, depression, aggression, anxiety, and hyperactivity but they may also contribute to the induction and/or maintenance of micturition disorders such as leakage, urge incontinence, and urinary retention. Therefore, non-invasive procedures, simple strategies such as parenteral education, bladder diary, constipation management, urotherapy, FMS, and pelvic floor rehabilitation seem to be the effective therapeutic options for AS associated with BBD and micturition disorders [16,17]. Numerous rehabilitative methods are currently available, which help minimize the negative impact of these functional impairments. In terms of voiding function, pelvic floor muscle exercise, biofeedback, FMS, neuromodulation, and clean intermittent self-catheterization are among the key rehabilitation strategies [18,19,20,21]. Given the lifelong implications of these dysfunctions, special attention must be paid to children to ensure timely intervention and improve long-term outcomes.

In the case presented in this study, we used FMS with good results. The method was well tolerated by our patient with AS and associated micturition disorders.

## 5. Conclusions

Autism spectrum disorder is an incapacitating disorder with significant impairment in social functioning. Psychological symptoms and other disorders frequently co-occur with ASD. Additionally, children with ASD are at greater risk of being affected by different forms of incontinence and LUTS. Therefore, screening for incontinence and, if indicated, the treatment of these disorders is recommended. Non-invasive procedure simple strategies such as parenteral education, bladder diary, the treatment of constipation, urotherapy, functional magnetic stimulation using Magneto Stym, and pelvic floor rehabilitation seem to be the effective therapeutic option for managing micturition disorders associated with ASD.

## Data Availability

The data presented in this study are available on request from the corresponding author. The data are not publicly available due to restrictions.
